# Supplementary material for: Validity of the Swimming Capacities and Anthropometric Indices in Predicting the Long-Term Success of Male Water Polo Players: A Position-Specific Prospective Analysis over a Ten-Year Period
Source: Int J Environ Res Public Health. 2022 Apr 7;19(8):4463. doi: 10.3390/ijerph19084463 (PMC9032409; doi:10.3390/ijerph19084463)
Supplement: Supplementary file 1 [file ijerph-19-04463-s001.zip › ijerph-1649275-supplementary.pdf]

Supplementary Table S1: Descriptive statistics for the total sample of studied water polo players at study baseline.

	Valid N	Mean	Minimum	Maximum	Std.Dev.
Body height (cm)	85	186.30	173.00	204.60	6.07
Body mass (kg)	85	84.82	63.00	112.00	9.65
BMI (kg/m^2)	85	24.40	18.62	32.03	2.13
Arm span (cm)	85	194.10	177.50	212.50	7.43
Body fat (%)	85	18.49	10.64	25.43	2.87
25mFS (s)	85	13.04	11.15	15.03	0.74
15mWS (s)	85	8.99	7.60	10.47	0.50
100mFS (s)	85	61.71	54.01	72.72	3.51
400mFS (s)	85	298.34	259.00	376.00	19.75
